# Design for a Highly Stable Laser Source Based on the Error Model of High-Speed High-Resolution Heterodyne Interferometers

**DOI:** 10.3390/s20041083

**Published:** 2020-02-17

**Authors:** Hongxing Yang, Ziqi Yin, Ruitao Yang, Pengcheng Hu, Jing Li, Jiubin Tan

**Affiliations:** Key Lab of Ultra-Precision Intelligent Instrumentation (Harbin Institute of Technology), Ministry of Industry and Information Technology, Harbin 150001, China; takeon@126.com (H.Y.); angelababy0811@hit.edu.cn (Z.Y.); hupc@hit.edu.cn (P.H.); 18S101084@stu.hit.edu.cn (J.L.); jbtan@hit.edu.cn (J.T.)

**Keywords:** beat frequency, drift, error model, heterodyne interferometer

## Abstract

Heterodyne interferometers with two opposite Doppler shift interference signals have been proposed for high-resolution measurement with high measurement speed, which can be used in the background of high-speed high-resolution measurement. However, a measurement error model for high-speed high-resolution heterodyne interferometers (HSHR-HIs) has not yet been proposed. We established a HSHR-HI measurement error model, analyzed the influence of beat frequency stability with a simplified optical structure, and then designed an offset-locked dual-frequency laser source with a digital control system to reduce the impact of beat frequency drift. Experiments were used to verify the correction of the measurement error model and the validity of the laser source. The results show that the new laser source has a maximum beat frequency range of 45 MHz, which shows the improvements in the measuring speed and resolution.

## 1. Introduction

Ultra-precision measurement and positioning provides essential support to modern precision engineering, lithography, and advanced scientific applications. Due to their robustness and direct traceability, heterodyne interferometers are widely applied in these fields to accurately measure displacements. Measurement with picometer resolution and a measurement speed of several meters per second is the general tendency in lithography and other precision engineering fields. However, the task of simultaneously achieving high resolution together with high-speed measurement is challenging due to several limitations. For example, periodic nonlinearity is one of the main errors restricting the measurement accuracy and resolution, originating from optical mixing and imperfect polarizing optical devices [1,2,3,4,5,6]. To overcome the issue of nonlinearity, methods from different research fields have been proposed [6,7,8,9]. Some researchers designed a balanced optical path interferometer to reduce the nonlinearity error; however, the measurement speed is restricted and may decrease by half when applied on a double-path interferometer [5]. This situation was not improved until a high-speed high-resolution heterodyne interferometer (HSHR-HI) with two opposite Doppler shift interference signals was proposed [10,11,12]. However, the measurable speed is restricted by the beat frequency (frequency difference) of the laser source [13,14,15]. ZYGO 7714 (Zygo, Middlefield, CT, USA) with AOM (acoustic optical modulator) can achieve spatially separated beams output with frequency differences of 20 MHz ± 1600 Hz, but the frequency difference was fixed to 20 MHz due to the enormous cost of the whole system [16].

Some studies were conducted to improve the output power of the laser while maintaining the high stability of the beat frequency. For example, a dual-frequency laser source with a phase locking loop technique was proposed, but the beat frequency was restricted to 8.5 MHz [17,18]. Large frequency offset stability is poor compared with small frequency offset stability due to hardware limitations and the frequency stabilization algorithm used in the design. Therefore, to improve productivity, the beat frequency of the laser source must be increased to improve the measurement speed.

The factors affecting the measurement accuracy of HSHR-HI are different from traditional interferometers because two opposite Doppler shifts are generated in both reference and measuring interference signals in the proposed interferometer. The measuring wavelength stability and the beat frequency stability are important to the measurement because they affect the signal processing and can deteriorate the measurement result. Therefore, a new measurement error model should be established and analyzed to guide the establishment of a measurement system.

In this study, a HSHR-HI measurement error model was established. Factors that can impact the measurement accuracy and measuring speed were analyzed, especially factors that are different from those affecting traditional interferometers like the stability of beat frequency. To reduce the influence of the beat frequency stability on the measurement accuracy, an offset-locked dual-frequency laser source with spatially separated output beams was developed, and experiments verified the correctness of the error model and the superiority of the laser source.

## 2. HSHR-HI Measurement Error Model 

### 2.1. HSHR-HI Measurement Principle 

**Figure 1:**
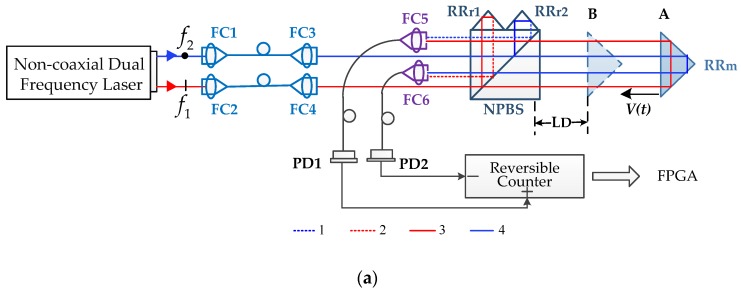

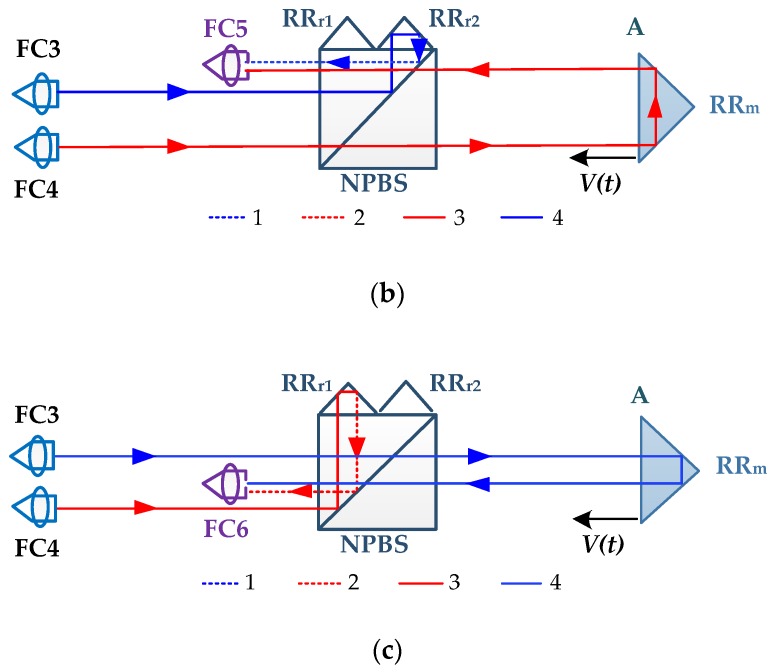
Schematic of the main composition of HSHR-HI (high-speed high-resolution heterodyne interferometer). FC, fiber coupler. NPBS, non-polarizing beam splitter; RR_r_, retro reflectors for the reference beams; RR_m_, retro reflectors for the measurement beams; PD, photodetector. (**a**) Overall optical configuration, (**b**) optical path of beams received by FC5, and (**c**) optical path of beams received by FC6.

The schematic of the main configuration of the HSHR-HI is shown in Figure 1. The orthogonal polarized dual frequencies *f*_1_ and *f*_2_ are generated from the laser source and transferred into the interferometer by using spatially separated optical fibers. The beams output from the fibers split into four beams in the interferometer. Beam 1 is reflected by specially prepared retro reflectors (RR) RRr1, combined with beam 3 reflected by retro reflectors RR_m_, and coupled into fiber coupler 5 (FC5) to generate beat signal IM (measurement signal); beam 2 is reflected by specially prepared retro reflectors (RR) RRr2, combined with beam 4 reflected by retro reflectors RR_m_, and coupled into FC6 to generate beat signal IR (reference signal), shown in Figure 1.

When the target moves at a speed of *υ*, a Doppler frequency shift (DFS) is generated. The signals *I*_M_ and *I*_R_ can be expressed as

(1) $$\begin{array}{l} I_{M}\propto \cos[2\pi (f_{1}+\Delta f_{d1})t]\cos(2\pi f_{2}t)\\ \;\propto \cos[2\pi (f_{b}+\Delta f_{d1})t] \end{array}$$

(2) $$\begin{array}{l} I_{R}\propto \cos(2\pi f_{1}t)\cos[2\pi (f_{2}+\Delta f_{d2})t]\\ \;\propto \cos[2\pi (f_{b}-\Delta f_{d2})t] \end{array}$$

where: $f_{b}$ ($f_{b}=f_{1}-f_{2}\approx 10^{7}\mathrm{Hz}$) is the beat frequency; $\Delta f_{d1}=2nvf_{1}/c$ and $\Delta f_{d2}=2nvf_{2}/c$ are the Doppler frequency shift due to the movement of RR_r1_ and RR_r2_, respectively; $f_{1}\approx f_{2}\approx 10^{14}\mathrm{Hz}$ and $2nv/c\approx 10^{-8}$, then $\Delta f_{d1}\approx \Delta f_{d2}\approx 10^{6}\mathrm{Hz}=\Delta f_{d}$. Therefore, the Doppler frequency shift $\Delta f_{d}$ between the reference and measurement signals is

(3) $$\Delta f_{d}=\frac{2n_{0}v}{c}f_{0}=\frac{2n_{0}v}{\lambda_{0}}$$

where $n_{0}$ is the nominal refractive index of air, $f_{0}$ is the nominal frequency of the laser source, and $\lambda_{0}$ is the wavelength of the laser beam in a vacuum. The DFS of two beam signals are equal numerically, but the signs are opposite to one other. Thus, the measurement resolution can be doubled if processing the phase difference of two signals using a reversible counter, which is received using a photoelectric detector for amplifying and shaping. The reversible counter *N*_0_ can be expressed as

(4) $$N_{0}=2\int_{t_{0}}^{t_{1}}\Delta f_{d}dt=2\int_{t_{0}}^{t_{1}}\frac{2n_{0}v}{\lambda_{0}}dt=\frac{4n_{0}L}{\lambda_{0}}.$$

The relationship between the displacement of target *L* and the output of reversible counter *N*_0_ is

(5) $$L=\frac{N_{0}\lambda_{0}}{4n_{0}}.$$

### 2.2. Measurement Error Model of HSHR-HI

In the ideal case, the coherent light signal generated from the laser source is

(6) $$E=ae^{i\left(2\pi ft+\varphi \right)}$$

where *a* is amplitude of the beam and φ is initial phase in the transmission. The frequency *f* in the equation is considered constant and does not contribute to displacement measurement. However, the real frequency of the beam varies within a certain range. The real light signal is rewritten as

(7) $$E=ae^{i[2\pi \int_{t_{{}_{0}}}^{t}f(t)dt+\varphi ]}.$$

When the measurement reflector RR_m_ moves from position A (considered the zero point) to position B in Figure 1, beams 1 and 3 can be expressed as

(8) $$E_{1}\left(t\right)=E_{1}(t-T_{r1})=a_{1}e^{i[2\pi \int_{t_{{}_{0}}}^{t-T_{r1}}f_{2}(t)dt+\varphi_{2}]}$$

(9) $$E_{2}\left(t\right)=E_{2}(t-T_{m1})=a_{2}e^{i\left\{2\pi \int_{t_{{}_{0}}}^{t-T_{m1}}\left[f_{1}(t)+\Delta f_{d1}(t)\right]dt+\varphi_{1}\right\}}$$

where: *T_m_*_1_ is the time delay caused by *L_m_*_1_ ($T_{m1}=n_{0}L_{m1}/c$), which is the spatial distance between FC4 and PD1; *T_r_*_1_ is the time delay caused by *L_r_*_1_ ($T_{r1}=n_{0}L_{r1}/c$), which is the spatial distance between the FC3 and the PD1; and $\Delta f_{d1}(t)=f_{1}(t)2n_{0}v(t+T_{m1})/c$ is the frequency shift due to the movement of RR_m_. Thus, the detected signal *I*_M_ can be expressed as

(10) $$\begin{array}{l} I_{M}(t)\propto E_{1}(t)\times E_{2}(t)\\ \;=a_{1}a_{2}e^{\{2\pi \int_{t_{0}}^{t-T_{m1}}\left[f_{1}(t)+\Delta f_{d1}(t)\right]dt-2\pi \int_{t_{0}}^{t-T_{r1}}f_{2}(t)dt+\varphi_{1}-\varphi_{2}\}.} \end{array}$$

The phase of the detected signal *I*_M_ is expressed as

(11) $$\varphi_{M}(t)=2\pi \int_{t_{0}}^{t-T_{m1}}\left[f_{1}(t)+\Delta f_{d1}(t)\right]dt-2\pi \int_{t_{0}}^{t-T_{r1}}f_{2}(t)dt+\varphi_{1}-\varphi_{2}.$$

Similarly, the phase of the signal *I*_R_ received by PD2 can be expressed as

(12) $$\varphi_{R}(t)=2\pi \int_{t_{0}}^{t-T_{r2}}f_{1}(t)dt-2\pi \int_{t_{0}}^{t-T_{m2}}\left[f_{2}(t)+\Delta f_{d2}(t)\right]dt+\varphi_{1}-\varphi_{2}$$

where *T_m_*_2_ is the time delay caused by *L_m_*_2_ ($T_{m2}=n_{0}L_{m2}/c$), which is the spatial distance between FC3 and PD2; *T_r_*_2_ is the time delay caused by *L_r_*_2_ ($T_{r2}=n_{0}L_{r2}/c$), which is the spatial distance between FC4 and PD2; and $\Delta f_{d2}(t)=f_{2}(t)2n_{0}v(t+T_{m2})/c$ is the frequency shift due to the movement of RR_m_. In a period of time $\left[t_{0}{,\;t}_{1}\right]$, the number of reversible counter output pulses is calculated as

(13) $$\begin{array}{l} N=\frac{1}{2\pi }\left\{\varphi_{M}(t_{1})-\varphi_{R}(t_{1})-\left[\varphi_{M}(t_{0})-\varphi_{R}(t_{0})\right]\right\}\\ \;=\int_{t_{0}-T_{m1}}^{t_{1}-T_{m1}}\Delta f_{d1}(t)dt+\int_{t_{0}-T_{m2}}^{t_{1}-T_{m2}}\Delta f_{d2}(t)dt+\int_{t_{1}-T_{r2}}^{t_{1}-T_{m1}}f_{1}\left(t\right)dt+\int_{t_{1}-T_{r1}}^{t_{1}-T_{m2}}f_{2}\left(t\right)dt-\int_{t_{0}-T_{r2}}^{t_{0}-T_{m1}}f_{1}\left(t\right)dt-\int_{t_{0}-T_{r1}}^{t_{0}-T_{m2}}f_{2}\left(t\right)dt \end{array}$$

where the frequency shift in the third term can be ignored due to *t* being about $10^{-9}$ to $10^{-7}$ s when $t\in [t_{1}-T_{r2},\;t_{1}-T_{m1}]$, which means $f_{1}(t)=f_{1}(t_{1})$, $t\in [t_{1}-T_{r2},\;t_{1}-T_{m1}]$. The frequency shift in the last three terms in Equation (13) can be ignored as well. Benefitting from the symmetrical optical configuration in Figure 1, the time delays in each path are the same as $T_{r1}=T_{r2}=T_{r}$, $T_{m1}=T_{m2}=T_{m}$. Accordingly, Equation (13) can be rewritten as

(14) $$\begin{array}{l} N=\int_{t_{0}}^{t_{1}}\frac{2n_{0}v(t)}{c}f_{1}(t-T_{m})dt+\int_{t_{0}}^{t_{1}}\frac{2n_{0}v(t)}{c}f_{2}(t-T_{m})dt\\ \;+f_{1}\left(t_{1}\right)\times \left(T_{r}-T_{m}\right)+f_{2}\left(t_{1}\right)\times \left(T_{r}-T_{m}\right)-f_{1}\left(t_{0}\right)\times \left(T_{r}-T_{m}\right)-f_{2}\left(t_{0}\right)\times \left(T_{r}-T_{m}\right) \end{array}.$$

Because $f_{1}(t)$ and $f_{2}(t)$ can be expressed as $f_{1}(t)\;=\Delta f_{1}(t)+\;f_{0}$ and $f_{2}(t)=\Delta f_{2}(t)+f_{0}$, respectively, where $\Delta f_{1}(t)$ and $\Delta f_{2}(t)$ are the differences between the real frequency and the nominal frequency, the first term in Equation (14) can be calculated as

(15) $$\int_{t_{0}}^{t_{1}}\frac{2n_{0}v(t)}{c}f_{1}(t-T_{m})dt=\int_{t_{0}}^{t_{1}}\frac{2n_{0}v(t)}{c}\left[\Delta f_{1}(t-T_{m})+f_{0}\right]dt=\frac{2n_{0}L}{\lambda_{0}}+\int_{t_{0}}^{t_{1}}\frac{2n_{0}v(t)}{c}\Delta f_{1}(t-T_{m})dt.$$

Because $T_{m}$ ($T_{m2}=n_{0}L_{m2}/c$) is in the range of $10^{-9}$ s, so $\Delta f_{1}(t-T_{m})\approx \Delta f_{1}(t_{0})=f_{1}(t_{0})-f_{0}$ and $\Delta f_{2}(t-T_{m})\approx \Delta f_{2}(t_{0})=f_{2}(t_{0})-f_{0}$. Then, the second term in Equation (15) can be calculated as

(16) $$\int_{t_{0}}^{t_{1}}\frac{2n_{0}v(t)}{c}\Delta f_{1}(t-T_{m})dt=\int_{t_{0}}^{t_{1}}\frac{2n_{0}v(t)f_{0}}{c}\times \frac{f_{1}(t_{0})-f_{0}}{f_{0}}dt=\frac{N_{0}}{2}\times \frac{f_{1}(t_{0})-f_{0}}{f_{0}}.$$

Similarly, the second term in Equation (14) can be expressed as

(17) $$\int_{t_{0}}^{t_{1}}\frac{2n_{0}v(t)}{c}\Delta f_{2}(t-T_{m})dt=\int_{t_{0}}^{t_{1}}\frac{2n_{0}v(t)f_{0}}{c}\times \frac{f_{2}(t_{0})-f_{0}}{f_{0}}dt=\frac{N_{0}}{2}\times \frac{f_{2}(t_{0})-f_{0}}{f_{0}}.$$

So, Equation (14) can be written as

(18) $$\begin{array}{c} N=\frac{4n_{0}L}{\lambda_{0}}+\frac{N_{0}}{2}\times \frac{f_{1}(t_{0})-f_{0}}{f_{0}}+\frac{N_{0}}{2}\times \frac{f_{2}(t_{0})-f_{0}}{f_{0}}\\ \;+\left[f_{1}\left(t_{1}\right)-f_{1}\left(t_{0}\right)\right]\times \frac{n_{0}}{c}(L_{r}-L_{m})+\left[f_{2}\left(t_{1}\right)-f_{2}\left(t_{0}\right)\right]\times \frac{n_{0}}{c}(L_{r}-L_{m}) \end{array}.$$

Therefore, the displacement measurement error can be calculated as

(19) $$\begin{array}{c} \Delta L\;=\frac{(N-N_{0})c}{4n_{0}f_{0}}\\ \;=\frac{f_{1}(t_{0})-f_{0}}{f_{0}}L-\frac{f_{1}\left(t_{1}\right)-f_{1}\left(t_{0}\right)}{f_{0}}L_{D}+\frac{f_{b}\left(t_{1}\right)-f_{b}\left(t_{0}\right)}{2f_{0}}L_{D}-\frac{f_{b}(t_{0})}{2f_{0}}L \end{array}$$

where $f_{b}(t_{1})$ is the beat frequency at *t*_1_, $f_{b}(t_{0})$ is the beat frequency at *t*_0_, and *L_D_* is the dead path length, $L_{d}=(L_{m}-L_{r})/2$. The fourth term in Equation (19), is much larger than the other three terms, so we used $f_{b}{}_{0}L/2f_{0}$ as a compensation to obtain

(20) $$\Delta L\;=\frac{f_{1}(t_{0})-f_{0}}{f_{0}}L-\frac{f_{1}\left(t_{1}\right)-f_{1}\left(t_{0}\right)}{f_{0}}L_{D}-\frac{f_{b}\left(t_{1}\right)-f_{b}\left(t_{0}\right)}{2f_{0}}L_{D}+\frac{f_{b}(t_{0})-f_{b0}}{2f_{0}}L$$

where $f_{b}{}_{0}$ is the nominal beat frequency of the laser. In Equation (20), the first term is the error related to the long-term frequency stability and movement distance of the target, the second term is the error related to the short-term frequency stability and dead path length, the third term is the error related to the short-term beat frequency stability and dead path length, and the fourth term is the error related to long-term beat frequency stability and movement distance of the target.

Shortening the dead path length can minimize the measurement error, and the long- and short-term frequency stabilities are relevant to the laser source, which many researchers have attempted to stabilize. However, the beat frequency stability is one aspect that has not yet been discussed, but is necessary to verify the impact of beat frequency drift on the measurement error and find a method to reduce the influence of the beat frequency drift on the measurement accuracy.

## 3. Experimental Validation

### 3.1. Validation for the Error Model of HSHR-HI

**Figure 2:**
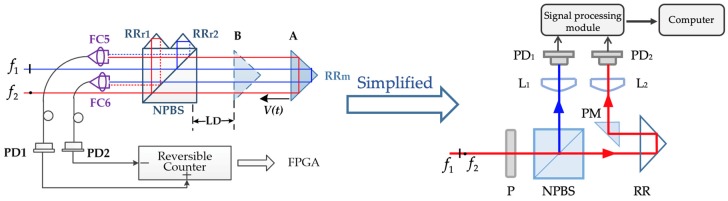
Scheme for the simplified optical path structure. P, polarizer; NPBS, non-polarizing beam splitter; RR, retro reflectors; PM, prism mirror; L, lens; PD, photodetector; M, mirror.

Analyzing the impact of beat frequency drift without other factors from Equation (20) is difficult using the optical structure shown in Figure 2. The displacement measurement error model was analyzed in Section 2.2 under ideal experimental conditions. In real measurements, however, some other factors affect the accuracy of measurement results, such as vibrations of the target and changes in the environment. The influence of vibration usually produces an error of tens of micrometers, changes in environment temperature usually produce an error of 0.12 nm/0.1 °C (21–22 °C), and the drift of beat frequency produces an error less than 1 nm [14,19]. This means that separating the error induced by the drift of beat frequency from the error induced by vibration and environmental change is hard. The error model in Equation (20) is based on the condition of a vacuum environment, but building or buying a large vacuum tank in which to place all the instruments would be costly. If the vacuum tank has a low vacuum degree, the measurement results would still be influenced by the change in the refractive index.

Therefore, spatially separated optical paths were changed into a common optical path structure to reduce the environmental influence, as shown in Figure 2. The beams with frequencies $f_{1}$ and $f_{2}$ pass through a polarizer and become an interfering beam. Then, this beam separates into two parts through NPBS: the reflected beam passes through a lens (L_1_) and then focuses on one photodetector (PD_1_) as the reference signal; the transmitted beam is reflected by a retro reflector (RR) and then redirects to the center of lens (L_2_) focusing on the photodetector (PD_2_). When the RR moves, frequencies $f_{1}$ and $f_{2}$ generate the same Doppler shift with the same environmental influence, so the signal received by PD_2_ is only related to beat frequency drift $f_{b}$ and optical path difference *L*. This simplified optical path structure can inhibit the impact of changes in the refractive index on the referring and measurement beams due to the common optical path. The beams in NPBS are refracted less, which can reduce ghost reflection error. The beam reflected by RR that directly proceeds into the PM and then received by PD_2_ could reduce the non-linear error compared with the complex optical structure. This structure could remove the influence of undesired variables other than the beat frequency drift in Equation (20).

In the experiment, the measurement and reference signals were analyzed by the signal-processing module, which was a custom-developed phasemeter with a frequency counter [20]. Its theoretical resolution at a measuring wavelength of 632.8 nm is about 4.8 pm. This simplified optical structure is similar to the optical structure analyzed by Chang et al. [21], assuming the optical length of the reference arm (laser–P–NPBS–PD_1_) is *l*_0_, the optical length of the measuring arm (laser–P–NPBS–RR–PM–PD_2_) is *l*_1_, so the signal received by photodetectors can be expressed as

(21) $$I_{r}=A\cos[(k_{2}-k_{1})l_{0}-2\pi f_{b}(t_{0})t]$$

(22) $$I_{m}=A\cos[(k_{2}-k_{1})l_{1}-2\pi f_{b}(t_{1})t]$$

where k=2π/λ=2πnf/c is the wave vector. Due to the optical length difference, $f_{b}=f_{2}-f_{1}$, $\Delta t=t_{1}-t_{0}=\frac{(l_{1}-l_{0})n}{c}\approx 10^{-9}s$. Thus, the measured phase difference is

(23) $$\varphi =\frac{2\pi n}{c}(f_{b}(t_{1})l_{1}-f_{b}(t_{0})l_{0})-2\pi (f_{b}(t_{1})-f_{b}(t_{0}))t$$

where n is the refractive index of air and *c* is the speed of light in the vacuum. Δt is so small that the phasemeter and the frequency counter can only collect the smoothing data due to the low sampling rate, so $f_{b}(t_{1})\approx f_{b}(t_{0})$. Equation (23) can be rewritten as

(24) $$\varphi =\frac{2\pi n}{c}f_{b}(t)L$$

where *L* ($L=l_{1}-l_{0}$) is the optical path difference. Then, the detected phase difference at moment t′ is

(25) $$\varphi \prime =\frac{2\pi n}{c}f_{b}(t\prime )L.$$

So, the measured phase difference caused by the drift of the beat frequency is

(26) $$\Delta \varphi =\varphi \prime -\varphi =\frac{2\pi n}{c}\Delta fL$$

where Δf is the drift of the beat frequency. Then, the position drift can be expressed as

(27) $$x=\frac{\Delta f}{f_{0}}\times L$$

where $f_{0}$ is the nominal frequency of the laser and *x* is the position drift calculated by the phasemeter.

The phase error of Equation (24) can be expressed as

(28) $$d\varphi =\frac{2\pi nL}{c}df_{b}+\frac{2\pi f_{b}L}{c}dn+\frac{2\pi nf_{b}}{c}dL$$

where: the drift of beat frequency ($f_{b}$) is less than 1 MHz in the experiment; *L* maintains a fixed value of 0.5 m in the experiment to validate the impact of the drift of beat frequency; when the target is static, the *dL* induced by minor vibrations in the environment is less than 100 nm; over a 0.1 °C temperature range, *dn* is 1 × 10^−7^ [19]. Assuming $f_{b}$ is 4 MHz and the temperature of the laboratory varies 0.1 °C, in Equation (28), the first to third terms are calculated as 10^−2^, 4 × 10^−9^, and 8 × 10^−8^, respectively, so the error that is introduced by the change in the air refractive index and the optical path difference can be ignored.

**Figure 3:**
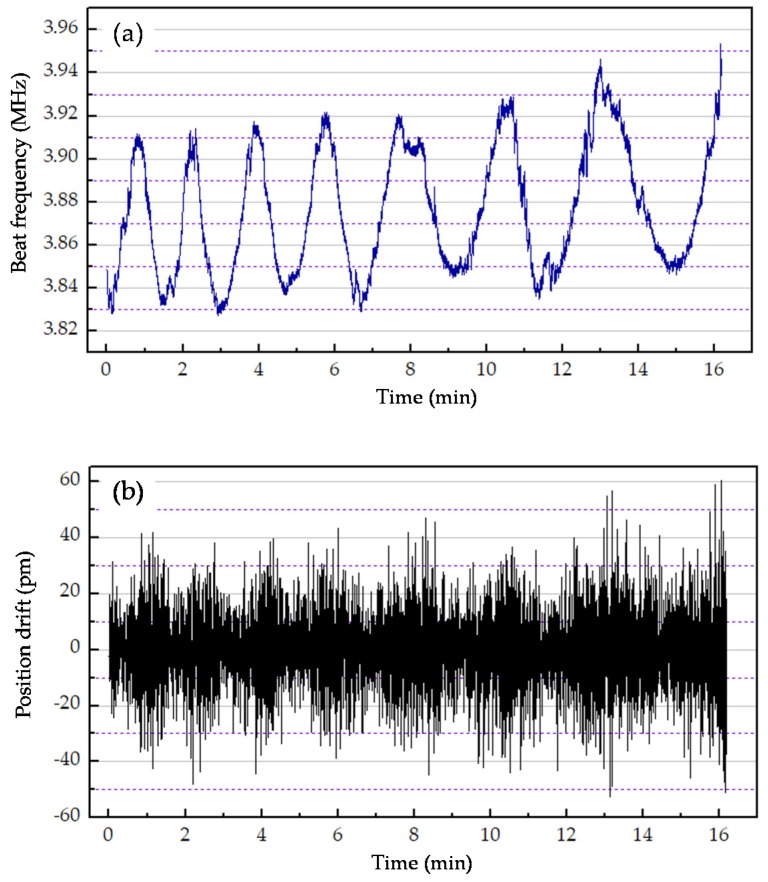
Scheme for the relationship between the beat frequency and the position drift: (**a**) beat frequency and (**b**) position drift.

Laser HIT 315 is a two-mode laser with a wavelength of 632.8 nm and adjustable frequency difference, which enabled accurate demonstration of the theoretical analysis in Equation (24). In the experiment, the PID (proportion integration differentiation) controller parameters of the laser were programmed to a large beat frequency drift condition—a drift of beat frequency more than 80 kHz/16 min. To determine the relationship between the beat frequency and the position drift simultaneously, a universal frequency counter (Agilent Technologies, Sabta Clara, CA, USA) was connected to the output of the reference photodetector with a BNC (bayonet nut connector) cable to collect the beat frequency signal, and the outputs of photodetectors were connected to the phasemeter to determine the value of position drift. The experimental result is illustrated in Figure 3.

From Equations (24)–(26), Δf can be expressed as:

(29) $$\begin{array}{c} \Delta f=f_{b}(t\prime )-f_{b}(t)=(f_{2}(t\prime )-f_{1}(t\prime ))-(f_{2}(t)-f_{1}(t))\\ \;=(f_{2}(t\prime )-f_{2}(t))-(f_{1}(t\prime )-f_{1}(t)) \end{array}.$$

According to the spectral line profile, $f_{2}(t\prime )-f_{1}(t\prime )$ can be positive or negative, as can $f_{2}(t)-f_{1}(t)$, so the sign of Δf can be positive or negative. The absolute value of Δf is determined by the beat frequency drift. As mentioned above, Δt was so small that the data collected by the phasemeter and the frequency counter collected were smoothed data rather than real-time data, which means the relationship of position drift and the beat frequency drift could not be depicted precisely in real time. Therefore, to identify the relationship between the position drift and the beat frequency drift, the peak-to-peak value in a period time can be used to more precisely depict this relationship. Figure 3 shows that the peak-to-peak value of the position drift is related with the peak-to-peak value of the beat frequency drift, which is consistent with the above theoretical analysis in Equation (24). In the first two minutes, the beat frequency of the laser source drifted 80 kHz, and the position drift agreed with the theoretical calculation, obtaining a maximum of 85 pm. As the beat frequency drift increased to 105 kHz, such as during the last four minutes of the experiment, the position drift increased to 110. Therefore, based on the above analysis, the beat frequency drift impacts the displacement measurement, and a laser source with less frequency drift must be developed to improve the measurement accuracy.

### 3.2. Setup for a Laser Source with Low Beat Frequency Drift

**Figure 4:**
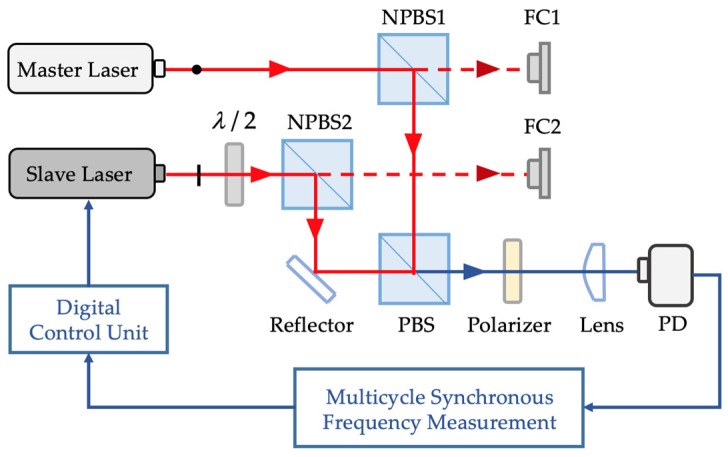
Experimental setup for the offset-locked dual-frequency laser source.

To improve the beat frequency stability, an offset-locked dual-frequency laser source with spatially separated output beams was developed. The offset-locked HSHR-HI laser source was designed and integrated as shown in Figure 4. The two incident beams from the master laser and the slave laser propagate through a half wavelength (λ/2) plate with perpendicular polarization. Then, each beam separates into two sub-beams through the non-polarizing beam splitter cube. Then, two sub-beams were used as the measurement beams separately coupled into the polarization-maintaining optical fiber by the fiber coupler (FC). The other two were used as the control beams that transmit into a PBS cube and a polarizer, received by the photoelectric detectors (PDs). The master laser and the slave laser used in the experiment were HIT 405, which is a kind of single-frequency laser with the stability of 1 × 10^−9^/24 h.

The two incident beams from the master laser and the slave laser propagated through a half wavelength (λ/2) plate with perpendicular polarization states. Then, each beam separated into two sub-beams by the non-polarizing beam splitter cube. Then, two of the sub-beams were used as the measurement beams coupled separately into the polarization-maintaining optical fiber by the FC; the other two sub-beams were used as the control beams transmitted into a PBS cube and a polarizer, received by the PDs. The master laser and the slave laser used in the experiment were HIT 405. 

**Figure 5:**
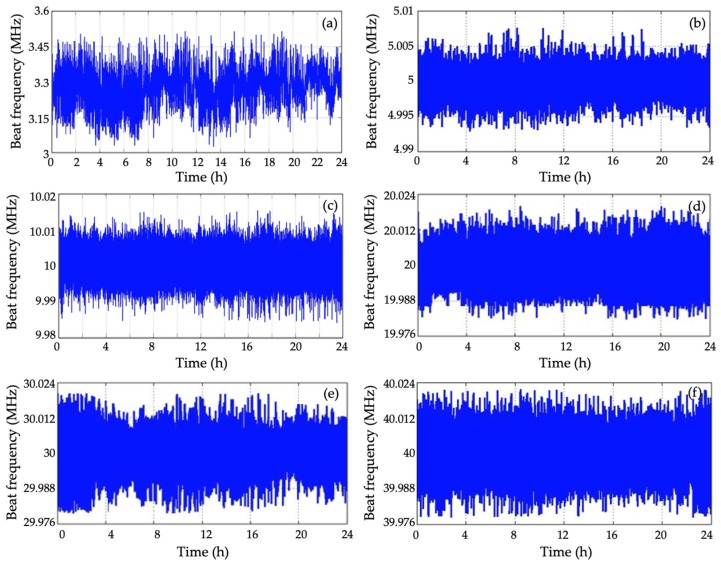
Beat frequency results over 24 h: (**a**) When the slave laser runs without offset-locked controlling, and when the offset-locked frequency is (**b**) 5, (**c**) 10, (**d**) 20, (**e**) 30, and (**f**) is 40 MHz.

To produce the desired beat frequency between the master and the slave lasers, the cavity length of the slave laser was changed by the electro-thermal drive circuit, which was controlled by comparing the result of the beat frequency to the desired beat frequency. The beat frequency stability of the master laser and slave laser were 500 kHz/24 h without digital control, as shown in Figure 5a.

To minimize the beat frequency drift, multi-cycle synchronization frequency measurement was used as a digital control system to process a large range of offset signals. The advantage of this method is that it can adjust the gate time signal period according to the offset frequency, which can properly allocate memory and improve the real-time performance of frequency measurement. The designed digital control system can measure the beat frequency range from 0.1 to 45 MHz with a 1 Hz resolution.

To testify the control ability of this digital system, the selected offset-locked frequencies were 5, 10, 20, 30, and 40 MHz, for which the beat frequency results over 24 h are shown in Figure 5.

**Figure 6:**
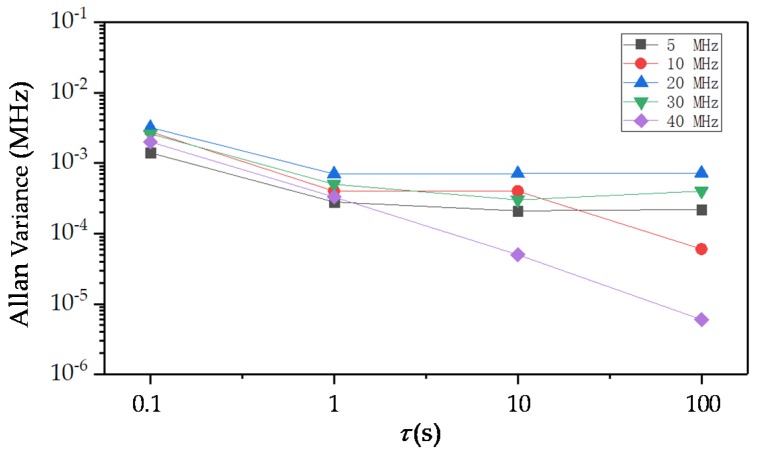
Allan variances at different τ values.

The beat frequency results in Table 1 show that the beat frequency drift was controlled within 40 kHz when the offset-locked frequency was set to 40 MHz. Figure 6 shows Allan variances at different τ values when offset-locked frequency was set to 5, 10, 20, 30, 40 MHz.

**Figure 7:**
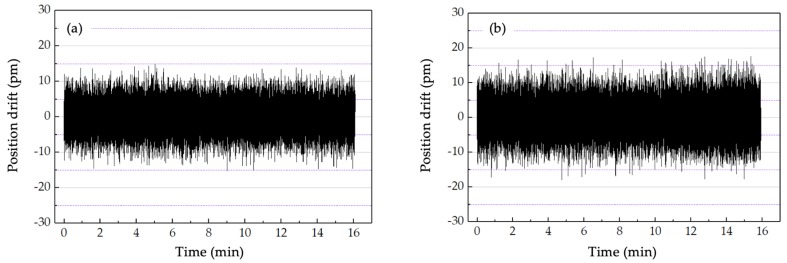
Position drift when the offset-locked frequency was set to (**a**) 10 and (b) 20 MHz.

**Figure 8:**
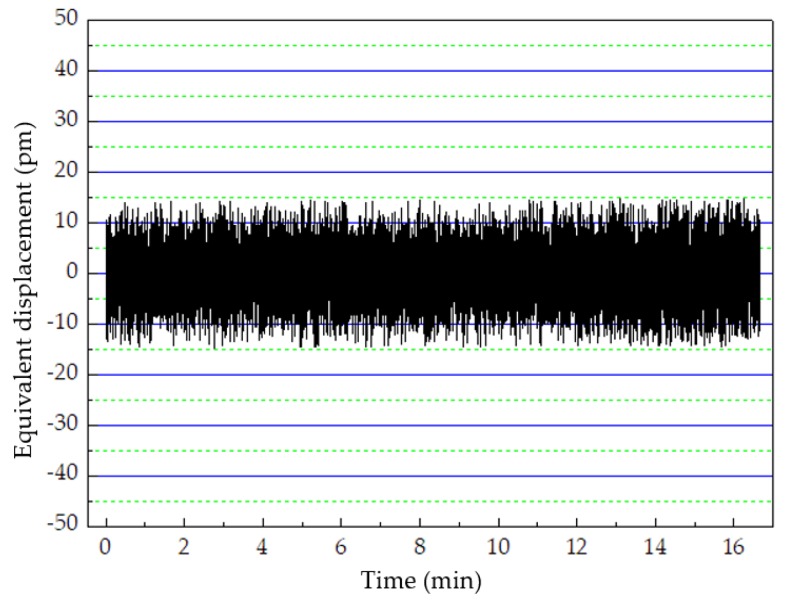
The noise impact on the measurement of position drift.

The next experiment was conducted to verify the position drift when the offset-locked frequency set to different values. The position drifts were 30 and 36 pm when the offset-locked frequency was set to 10 (28 kHz drift) and 20 (34 kHz drift), respectively (Figure 7). When the offset-locked frequency was set to 10 MHz, the results of position drift did not agree with the beat frequency drift in the theoretical analysis in Equation (24), mainly due to the circuit noise, which can mask the useful signal. To detect the impact of noise, an experiment was conducted in dim light, and the result is shown in Figure 8. As illustrated in Figure 8, the measurement error caused by the circuit noise in the system was 30 pm, which was mainly induced by the amplifiers in the circuits.

Additional experiment results show that the signals of position drift are inaccurate due to the phasemeter noise when the offset-locked frequency is lower than 10 MHz or higher than 20 MHz. The circuit noise that induces an error less than ±10 pm can mask the useful signal when the offset-locked frequency is lower than 10 MHz. When the offset-locked frequency is higher than 20 MHz, the high-frequency noise can impact the accuracy of the experiment result with an error less than ± 20 pm.

To compensate for the measurement error caused by the beat frequency drift, further research is needed to eliminate the circuit noise and the high-frequency noise. Although circuit noise produced a 30 pm measurement error in the experiment, the experiment results verified the impact of the beat frequency drift on measurement and showed the improvements in the measuring speed and resolution of the proposed new laser source.

## 4. Conclusions

In this study, we analyzed the influence of beat frequency stability on the HSHR-HI measurement error model. To reduce the impact of beat frequency drift, an offset-locked dual-frequency laser source with a digital control system was proposed. Experiments verified the correction of the measurement error model and the validity of the laser source. The results showed that the new laser source has a maximum beat frequency of 45 MHz, which improves speed measurement. Further studies should focus on reducing the beat frequency drift to hundreds of Hz and decreasing the noise of a phasemeter.

## Figures and Tables

**Table 1:** The beat frequency drift and the relative stability at different offset-locked frequencies.

Offset-Locked Frequency (MHz)	Time (h)	Frequency Center (MHz)	Peak-to-Peak Beat Frequency Drift (kHz)	Allan Variance (τ=1 s, kHz)
5	24	5.00109	12	0.3
10	24	9.99997	28	0.4
20	24	20.00021	34	0.7
30	24	30.00035	38	0.5
40	24	39.99996	40	0.3
